# How Race and Birthweight May Influence Gender Differences in Retinopathy of Prematurity

**DOI:** 10.3390/children13030324

**Published:** 2026-02-25

**Authors:** Robert W. Arnold, Jack Jacob

**Affiliations:** 1 Alaska Blind Child Discovery, Alaska Children’s EYE & Strabismus, 3500 Latouche #280, Anchorage, AK 99508, USA; 2 Alaska Neonatology Associates, Anchorage, AK 99508, USA; jackjacobalaska@gmail.com

**Keywords:** retinopathy of prematurity, male gender, neonatology screening

## Abstract

Background: ROP Check^®^ monitoring and documentation software from 28 American hospitals collected clinical data from 2010 to 2024 representing infants from a wide range of races and ethnicities. Methods: De-identified data compared gender to treatment status, race, birthweight (BW), gestational age (GA) and gestational age at first treatment. Results: From 7070 total patients, with 5060 having timely or early initial exams based on American Academy of Pediatrics (AAP) guidelines, 386 had treatment for ROP. Males constituted 54.3% of treated infants and 54.4% of all infants. There was no gender difference in gestational age or age at treatment, but males had greater birthweights (685 to 610 g). There were more females treated under 600 g. There were race-related birthweight differences in infants treated for ROP. Conclusion: There are more males screened and treated for ROP, but treatment rates are similar for both genders. Male preponderance reverses for infants with birthweight less than 600 g. Race has an influence on treated ROP.

## 1. Introduction

American neonatologists screen for retinopathy of prematurity (ROP) utilizing American Academy of Pediatrics (AAP) guidelines [1]. Treatment-warranted ROP is primarily influenced by gestational age (GA) and birthweight (BW) [2] but also other clinical variables, with race and gender as potential factors. Many studies have shown that males are over-represented in treatment-warranted ROP groups without additional details. However, when comparing screened patients by sex, no differences in treatment warranted ROP is found [3,4]. Yang et al. in 2006 [4] noted male gender (*p* < 0.005) in addition to non-black race (*p* < 0.0005) as risk factors for treatment-warranted ROP. In a large recent meta-analysis of 316 studies and 31,026 infants treated for ROP [3], males were over-represented in the treatment group (55% of those treated). However, a similar proportion of screened males and females were treated (*p* = 0.67). In 68 mixed-sex twin and multiple-delivery premature infants, there was no difference in advanced ROP stage by sex [5]. These findings can generally be explained by the greater incidence of prematurity, higher NICU admission rate, and major morbidity in male premature infants compared to female infants [6,7,8]. There are additional sex differences in ROP reported. Male infants with treatment-warranted ROP had greater birth weights and were treated earlier [5].

We have previously reported on the characteristics of race on treatment-warranted ROP in a large racially diverse dataset from ROP Check^®^ that includes all races as defined by the US Census Bureau. We reported racial differences in birthweight for treatment-warranted ROP but no sex-related differences across races. In this manuscript, we further investigated the characteristics of infants’ gender on treatment-warranted ROP.

The purpose of this study was to investigate the role of gender in addition to the previously reported role of gestational age and birthweight on predicting treatment-warranted ROP. We reviewed our dataset covering 2010 through 2024 from ROP Check^®^, a cloud-based monitoring and documentation software from 28 hospitals, to assess details on the impact of gender for premature infants over a range of birthweights with a treatment percentage of 7.6%. ROP Check^®^ functions to capture all infants meeting American Academy of Pediatrics (AAP) guidelines based on gestational age and birthweight without selection bias.

## 2. Methods

ROP Check^®^ (Neolight, Phoenix, AZ, USA) is a cloud-based EMR used for ensuring ROP exams occur in a timely manner, assisting neonatologists to conform to complex screening guidelines and aiding ophthalmologists tracking ROP staging exams and treatment [9]. We used a de-identified dataset of examination results from 28 hospitals using the software program from 2010 to 2024. The breakdown for the neonatal intensive care units (NICUs) was as follows: four Level 4 NICUs, six Level 3B NICUs, twelve Level 3 NICUs, and six Level 2 NICUs. The American Academy of Pediatrics ROP guidelines recommend all infants less than 31 weeks gestational age get ROP staging exams^1^. Surviving infants with birth gestational age of 21 + 6 weeks to 30 + 6 weeks were included in the analysis. IRB exemption (45 CFR § 46.104(d)(4),) was obtained for using the de-identified dataset from WCG IRB on 3/4/242. The data comply with the Declaration of Helsinki and the Health Insurance Portability and Accountability Act (HIPAA). Hospitals initiated the use of the program at different years. Race was recorded by NICU clinical staff and reflected infant’s race/ethnicity. Infant’s sex was determined by the neonatologist and was then recorded as male, female or ambiguous (intersex). Only one was eventually diagnosed with ambiguous genitalia and was excluded from the analysis.

Patient demographic data including neonatologist-determined sex, and race/ethnicity data were collected as initially entered when patients were clinically enrolled in ROP Check^®^. Clinical data birthweight (BW) and gestational age (GA) and gestational age at time of initial treatment from ROP Check^®^ were analyzed for normal distribution by Shapiro–Wilk test before non-parametric analysis. Data were analyzed with the non-parametric Mann–Whitney test, chi-square and power and logarithmic regressions, and receiver operating characteristic (ROC) curves for multivariable prediction.

## 3. Results

The ROP Check^®^ dataset had 7060 total infants. There were 5063 infants that had timely or early scheduled exams (at a post-menstrual age of 32 weeks or less) that were included in the analysis. Of these, 386 required anti-VEGF (85 female and 123 male) and/or laser (82 female and 96 male) therapy. The remaining 1997 patients underwent much of their NICU care at hospitals not using ROP Check^®^. These other infants were generally referred to hospitals using ROP Check^®^, with those with advanced ROP needing treatment, and these were excluded from the analysis.

**Figure 1:**
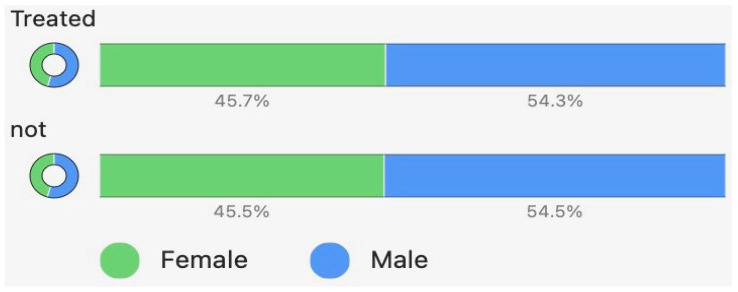
Proportion of male and female infants total and treated for ROP.

The distribution of male and female infants in the treated and untreated infant groups is demonstrated in Figure 1. There were significantly more males screened for ROP (*t*-test z = 6.4, *p* < 0.001) and treated for ROP (*t*-test z = 2.65, *p* = 0.008). The percentage of treated infants compared to the non-treated infants was similar for male and female infants, indicating that males were not intrinsically at greater risk for treatment (chi-square z = 0.91, *p* = 0.36).

**Figure 2:**
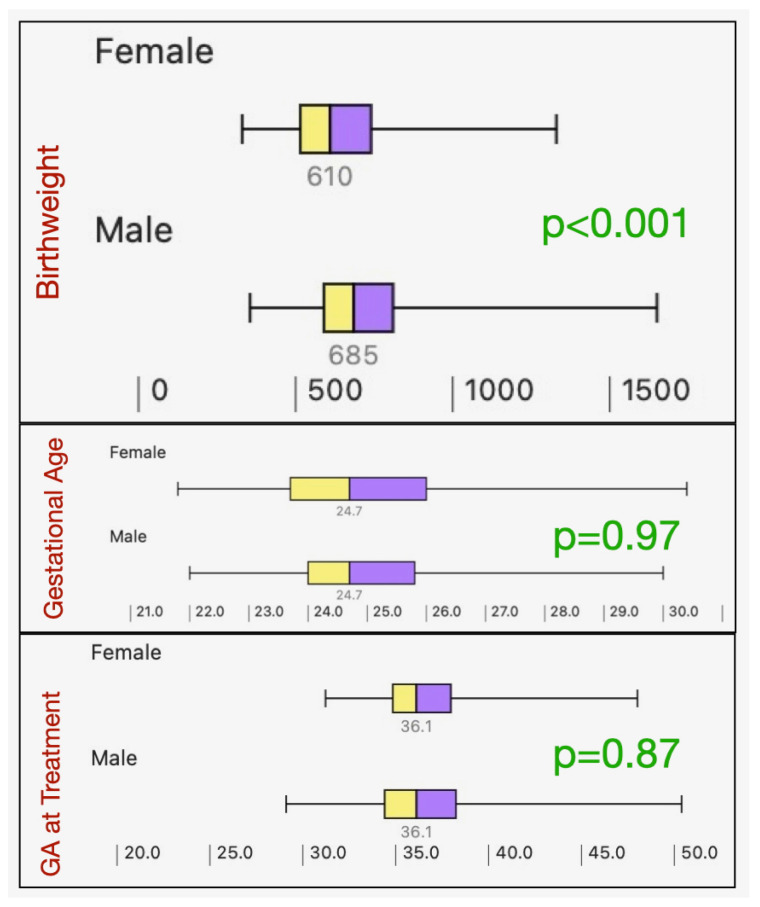
Boxplots showing gender influence of birthweight (grams), gestational age (weeks) and the gestational age at first ROP treatment (weeks) with *p*-values for Mann–Whitney statistics.

Male and female infants were further compared on the basis of gestational age, birthweight and gestational age at time of treatment (Figure 2). There was no gender difference for the full group in terms of gestational age (Mann–Whitney z = 0.61, *p* = 0.54) or gestational age at time of first treatment (Mann–Whitney z = 0.07, *p* = 0.94). However, males had significantly greater birthweight than the female infants (Mann–Whitney z = 5.9, *p* < 0.001).

**Figure 3:**
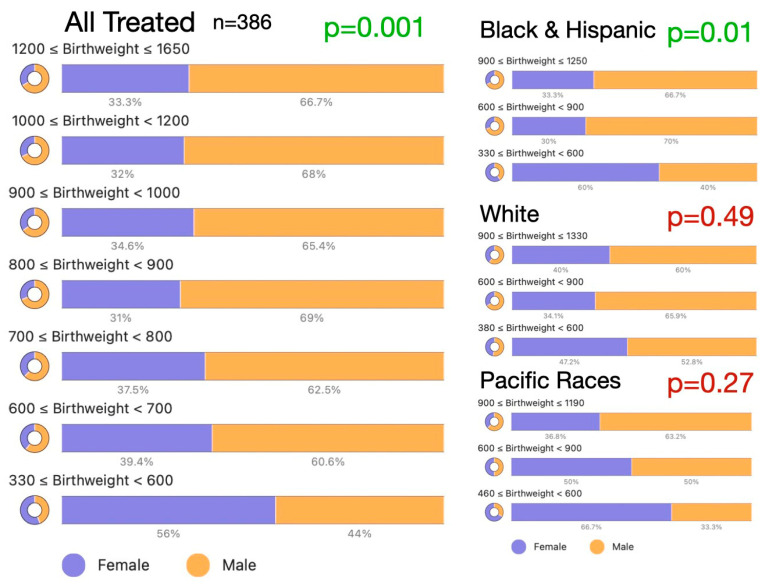
Influence of race/ethnicity and birthweight on gender distribution for infants treated for retinopathy of prematurity in the ROP Check dataset 2010–2024.

Figure 3 shows the influence of race/ethnicity and treatment status on the birthweight distribution of gender in patients. Table 1 shows the chi-square data comparing birthweight and gestational age influence of treated and all patients. Although the relationship between gestational age and birthweight has been previously well-documented (BW = 30.8 e^(0.126 GA)^, R^2^ = 0.63 for our dataset), we found remarkably different influences of gender in these premature infants. The preponderance of males was limited to the higher birthweights in most racial groups but reached statistical significance in infants of Black race, infants of Hispanic ethnicity and patients classified as “other.” High-risk Pacific race infants did not have a particularly strong influence on male preponderance in ROP (chi-square X^2^(4) =3.4, *p* = 0.5 treated infants, and X^2^(4) =7.8, *p* = 0.1 for the whole dataset). This suggests that birthweight differences reported in males vs. females treated for ROP may be influenced by race.

**Figure 4:**
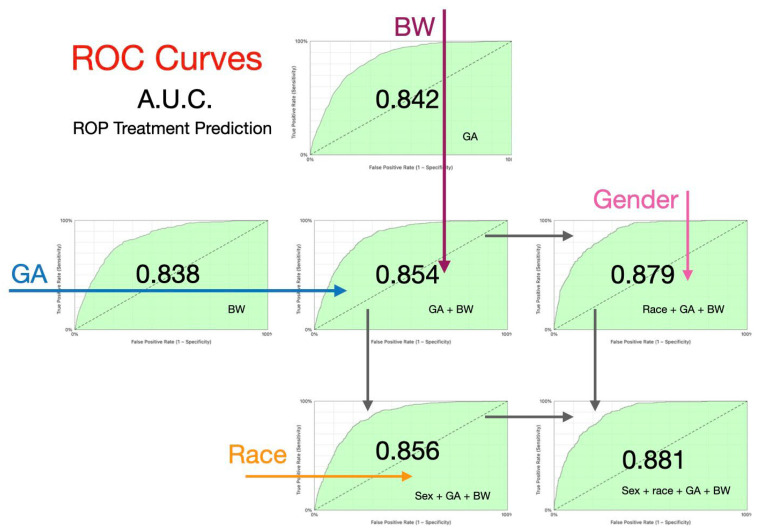
Influence of risk factors (arrows) on a predictive model for need for ROP treatment including gestational age (GA), birthweight (BA), race/ethnicity and gender. Receiver operating characteristic (ROC) curves shown with area under the curve (AUC) labeled in the center with each additional risk factor.

A model predicting treatment for ROP showed expected significant influence of gestational age (area under the curve (AUC) for receiver operating characteristic (ROC) curve 0.842) even higher than for birthweight (AUC 0.838). Combining gestational age and birthweight increased prediction (AUC 0.854; Figure 4). Added to those recognized screening risk factors, patient race/ethnicity increased prediction further (AUC 0.879). Gender added modestly to AUC for gestational age, birthweight and race (AUC 0.881, *p* = 0.011).

**Figure 5:**
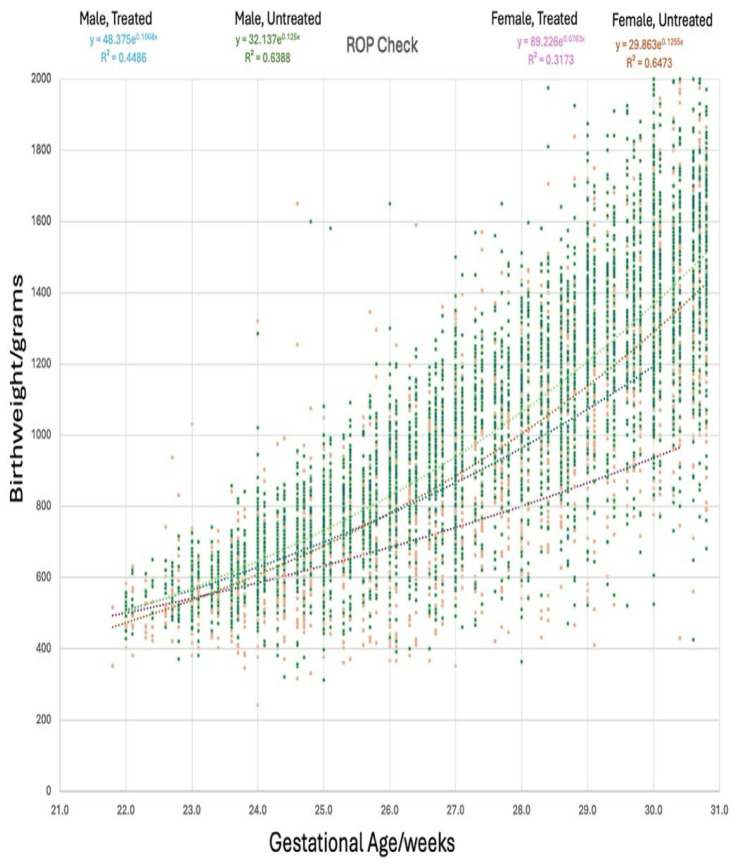
The influence of gender on the relationship between gestational age and birthweight separated by gender for data from ROP Check 2010–2024. Polynomial highly significant regression fits for all male infants (green), all female infants (orange), and all treated male infants (blue) are similar; however, all treated female infants (pink) had polynomial regression resembling small-for-gestational-age infants.

The relationship between gestational age and birthweight for male and female premature infants with or without Type 1 ROP is shown in Figure 5 with highly significant second-order polynomial regressions. These regressions were very similar for all males and females screened and for treated males. However, the treated females had lower birthweights for gestational age resembling small-for-gestational-age infants.

**Figure 6:**
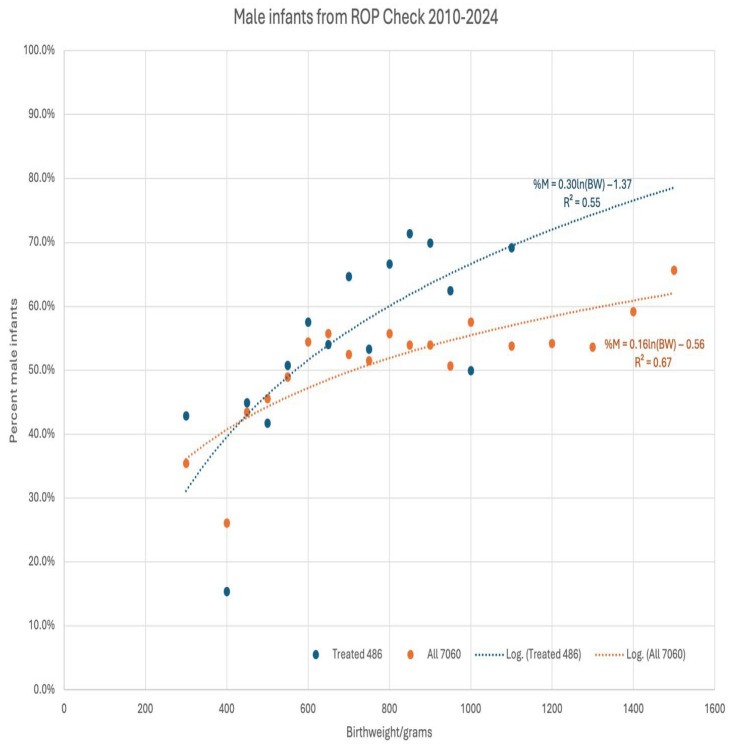
The percent of male infants as a function of birthweight and treatment status divisions with at least *n* = 10. The regression fits are indicated by blue and orange dotted lines with logarithm regressions and correlation R^2^ values. Infants with birthweight greater than 600 g were predominantly male, while those less than 600 g were predominantly female.

The birthweight influence on gender in ROP was evaluated by regressing the proportions of male infants against 50 major subsets for all 7060 infants and the 486 treated infants (Figure 6). For the timely and early (*n* = 5063) infants, there was a consistent influence of gender with an increase in male preponderance, with increasing birthweight, with treatment (X^2^(5) = 15, *p* = 0.01), and without treatment (X^2^(5) = 52, *p* < 0.001). However, for infants below 600 g, there was a consistent female preponderance suggesting a survival advantage for females. Of all infants less than 600 g birthweight, females with no treatment numbered 168, anti-VEGF 55 and laser numbered 21, while males with no treatment numbered 118; those with anti-VEGF 40 and males treated with laser numbered 19.

**Figure 7:**
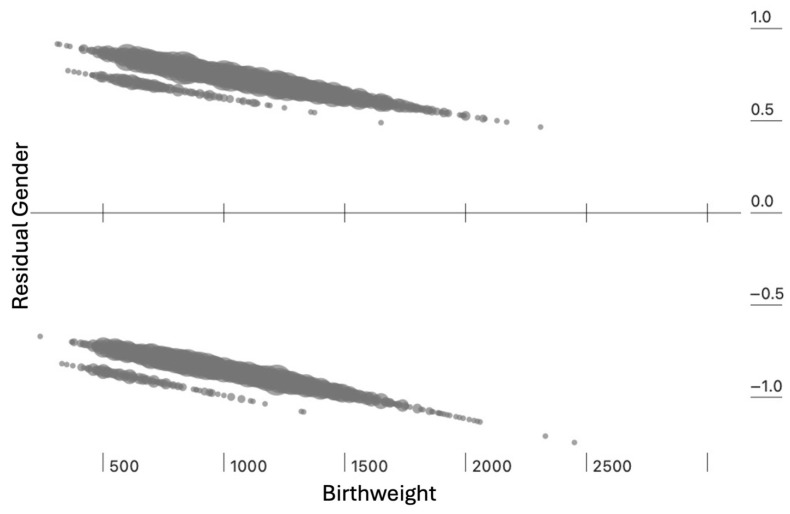
Significant multivariable regression influence on gender with residuals for birthweight (*p* < 0.001) and treatment status (*p* = 0.003).

For the 5042 timely- and early-screened infants, multivariable regression found significant influence on gender by birthweight (beta 0.00038 ± 0.000056, *p* < 0.001) and treatment status (beta −0.21 ± 0.07, *p* = 0.003), with residuals demonstrated in Figure 7.

## 4. Discussion

Our study represents the most comprehensive investigation on the effect of an infant’s sex on treatment-warranted ROP. Some of our findings confirmed what others have found: males are overrepresented in the population screened for ROP, but the incidence of treatment-warranted ROP does not differ between males and females (2). Males have been known to be overrepresented in the NICU preterm population, and this is likely the reason for their overrepresentation in the screened population [8]. The fact that the incidence of treatment-warranted ROP in the screened population is similar for males and females indicates that males and females do not differ in their risk for developing treatment-warranted ROP. This is also substantiated in animal models [10].

From a robust, large meta-analysis, 50 of the 316 studies found similar gestational age (27.5 vs. 27 weeks) and gestational age at first treatment (36 vs. 35.3 weeks) and birthweight (1028 vs. 955 g) for male and female infants [3]. Our results also confirm that there are no differences between males and females in gestational age at birth and gestational age at first treatment. Our study, however, differed from the meta-analysis in that our treated females had lower birthweight than males. The meta-analysis did not further break down the influence of birthweight on gender. A possible explanation of the different birthweight findings may be explained by the substantially different birth gestational ages in the meta-analysis and our study. The gestational age at birth in our cohort differed rather substantially from the meta-analysis (27.5 weeks for males and 27 weeks for female infants compared to 24.7 weeks for both males and females in our study). The fact is that gender differences for higher NICU mortality and morbidity in males disappear at later gestational ages. This emphasizes the fact that females have a survival advantage at earlier gestational ages and lower birthweights [8].

Utilizing the high-risk ROP-Check^®^ dataset, we confirmed the male preponderance in ROP both for premature infants and for those treated for ROP that has previously been reported [4]. The male infants had higher birthweight despite equal gestational age. Our study adds the finding that the male preponderance reverses for infants weighing less than 600 g at birth. This is likely due to the relative survival advantage for female infants admitted to the NICU. Generally, male infants admitted to the NICU have a higher incidence of death and morbidity, which may explain these differences [6,11].

The fact that we found significant birthweight differences in certain racial groups (Black and Hispanic) and not in the high-risk Pacific race group has implications for studies on gender differences in the incidence of ROP and treated ROP. Our ROP-Check^®^ dataset is unique in that it includes a substantial number of infants of Pacific races (American Indian/Alaska Native, Asian and Pacific Islander) that are at high risk for treatment-warranted ROP [12,13,14]. We have previously reported that African American infants treated for ROP have a lower birthweight while American Indian/Alaska Native and Pacific Islander infants have a higher birthweight [12]. These racial differences may explain some of the conflicting findings related to gender risk for ROP, since the racial characteristics of the study population may affect results. It is our conclusion that studies on the influence of race on American ROP treatment should address a complete racial profile as defined by the US Census Bureau in their population that include both low-risk (African American) and high-risk (Pacific races) in addition to intermediate-risk (Caucasian and Hispanic) factors [15]. These issues are also important when developing ROP screening guidelines internationally.

Strengths of this study include a large, U.S. academic and private hospital database over a decade that includes a wide distribution of racial and ethnic premature infants. Weaknesses include lack of photographic retinal image documentation and comparison to determine threshold for therapy of a portion of the infants.

## 5. Conclusions

We detected a strong birthweight-related increase in male infants over most of our dataset without influence of gestational age; however, we also found that the preponderance of infants with birthweight less than 600 g was mostly female. Race affects gender distribution in our infants treated for ROP.

## Figures and Tables

**Table 1:** Chi-square influence of birthweight (quintiles) on gender by race for all infants or those treated from the ROP Check^®^ database. Pacific race includes Asian, Pacific Islanders and American Indian/Alaska Native infants. *p* represents probability.

	Race	Treat?	*n*	X^2^(4)	*p*
**Birthweight**	All	treated	386	12.8	0.01
		all	5053	38	<0.001
	Black	treated	60	7.02	0.07
		all	1022	10.9	0.03
	Hispanic	treated	46	6.2	0.04
		all	533	7.2	0.13
	Pacific	treated	59	4.3	0.23
		all	349	5.2	0.27
	White	treated	90	3.37	0.50
		all	805	7.76	0.10
	Other—Missing	treated	130	10.5	0.03
		all	2330	31.6	<0.001
**Gestational Age**	All	treated	386	7.44	0.11
		all	5053	3.55	0.47
	Black	treated	60	2.94	0.40
		all	1022	2.69	0.61
	Hispanic	treated	46	1.92	0.59
		all	533	1.74	0.78
	Pacific	treated	350	3.19	0.53
		all	59	3.94	0.41
	White	treated	90	5.53	0.24
		all	805	1.01	0.91
	Other—Missing	treated	130	8.7	0.07
		all	2330	4.77	0.31

## Data Availability

Please contact the authors. Data Access: https://www.abcd-vision.org/references/ROPrace2025.pdf. Accessed on 24 February 2026.

## Abbreviations

The following abbreviations are used in this manuscript:

NICU	Neonatal intensive care unit
ROP	Retinopathy of prematurity
AUC	Area under the curve
ROC	Receiver operating characteristic
